# The STAT3‐CCND2 Axis Drives a Proliferative Metaplastic Precursor Population in Gastric Intestinal Metaplasia

**DOI:** 10.1111/jcmm.71283

**Published:** 2026-07-15

**Authors:** Fazhan Li, Huijuan Wen, Feifei Ren, Pengyuan Zheng, Simeng Liu

**Affiliations:** ^1^ Henan Key Laboratory for Helicobacter Pylori and Digestive Tract Microecology, the Fifth Affiliated Hospital of Zhengzhou University Zhengzhou University Zhengzhou Henan China; ^2^ Department of Gastroenterology The Fifth Affiliated Hospital of Zhengzhou University Zhengzhou Henan China

**Keywords:** DNA methylation, gastric intestinal metaplasia, gastric organoids, single‐cell RNA sequencing, transcriptomic sequencing

## Abstract

Gastric intestinal metaplasia (GIM) is a crucial precancerous lesion with ill‐defined drivers, and identifying regulators of its early proliferation and reprogramming is key to interception. We integrated epigenomic‐transcriptomic analysis of human GIM and normal tissues, validated targets via dual‐luciferase assay, and mapped cellular heterogeneity by single‐cell RNA sequencing of patient‐derived gastric organoids. Functional validation using organoids included STAT3 knockdown assessed by qRT‐PCR and immunofluorescence. STAT3 was identified as a top regulator of hypomethylated, upregulated genes in GIM and directly transactivated CCND2. Single‐cell analysis revealed a dominant proliferative “Cycling GIM‐precursor” population co‐expressing STAT3/CCND2. STAT3 knockdown reduced CCND2 and intestinal markers (CDX2, MUC2) in GIM organoids. The STAT3–CCND2 axis is central to early GIM pathogenesis, offering targets for early detection and chemoprevention of gastric cancer.

**Abbreviations**
CSCs: cancer stem cells
DEGs: differentially expressed genes
DEGs: differentially expressed genes
DMGs: differentially methylated genes
FPKM: fragments per kilobase of transcript per million mapped reads
GC: gastric cancer
GC: gastric cancer
GEO: Gene Expression Omnibus
GIM: gastric intestinal metaplasia
GIM: gastric intestinal metaplasia
scRNA‐seq: single‐cell RNA sequencing
TFs: transcription factors

## 1 Introduction

Globally, gastric cancer (GC) continues to pose a significant health challenge, representing the fifth most prevalent neoplasm and the fourth highest contributor to cancer‐associated fatalities [1]. Intestinal‐type GC, accounting for the vast majority of GC, primarily develops through the Correa cascade [2]. The pathogenesis typically evolves through a multi‐step sequence: initial chronic active gastritis (commonly associated with *Helicobacter pylori* infection), progressing to atrophic gastritis, then to gastric intestinal metaplasia (GIM), subsequently to dysplasia, and culminating in invasive carcinoma. Within this continuum, GIM is considered a critical transitional lesion [3]. Native gastric mucosa undergoes a fundamental identity shift, losing its characteristic cell types, such as parietal and chief cells, and acquiring intestinal phenotype cells at this stage [4]. Intestine‐specific transcription factors such as caudal‐type homeobox 2 (CDX2) and structural proteins such as mucin 2 (MUC2) are abnormally expressed in gastric epithelial cells in GIM [5]. Consequently, finding the molecular drivers responsible for the initiation and maintenance of GIM is important for developing effective chemopreventive strategies.

The cause of GIM is multifactorial. *H. pylori* infection serves as the primary environmental driver, with bacterial virulence factors such as CagA and VacA inducing chronic inflammation, oxidative stress, and epithelial cell damage [6]. However, a significant clinical problem is that metaplastic changes willl be persisted even if *H. pylori* is successfully eradicated in some patients. This observation strongly implies that self‐sustaining intracellular reprogramming mechanisms may occur in epigenetical level during the chronic inflammatory phase, enabling the metaplastic phenotype to maintain autonomously [7]. In this context, epigenetic alterations, particularly aberrant DNA methylation, have emerged as pivotal early events in carcinogenesis [8]. Hypermethylation of CpG islands in promoter regions is a well‐established mechanism for the transcriptional silencing of tumour suppressor genes [9, 10]. Conversely, global or locus‐specific hypomethylation could lead to the activation of oncogenes, retrotransposons, and some cancer‐associated signalling pathways [11]. Despite the above is known, conventional bulk tissue analyses obscure the cellular heterogeneity inherent in the gastric mucosa, which is a complex ecosystem comprising diverse epithelial, stem, progenitor and immune cells. This limitation makes it difficult to confirm that epigenetic changes originate from the stem cells, committed progenitors or differentiated cells and how they specifically alter cell fate. Besides, studies have shown that DNA methylation could inhibit the binding of transcription factors (TFs), thereby downregulating gene expression [12]. However, the epigenetic modifications of GIM and the mechanism by which transcription factors regulate GIM‐associated target genes are still unclear.

In our study, we integrated matched transcriptome and methylome datasets from human GIM and normal gastric organoids to identify a core set of epigenetically “derepressed” driver genes. We projected these genes onto a high‐resolution single‐cell transcriptomic atlas of the gastric mucosa to resolve their cell type‐specific expression patterns and dynamics during lineage plasticity. Moreover, dual luciferase, IF and qRT‐PCR experiments were performed to prove whether STAT3 could directly transactivate CCND2 to drive cell proliferation and positively regulate the expression of core intestinal metaplasia markers such as CDX2 and MUC2. To further support this thesis, the expression of pSTAT3 and CCND2 across the Correa cascade and in *H. pylori* ‐infected GES‐1 cells model, and that of stemness markers SOX9 and LGR5 in GIM organoids upon *STAT3* knockdown were examined. In summary, our study aims to provide new insights into the pathogenesis of GIM and offer the possibility for its reversal.

## 2 Materials and Methods

### 2.1 Data Acquisition and Preprocessing

Publicly available transcriptomic and DNA methylation datasets for GIM and normal organoids were retrieved from the Gene Expression Omnibus (GEO) under accession number GSE210995 [13]. The dataset includes transcriptome sequencing, methylation sequencing, and single‐cell data derived from GIM organoids (28 samples in total) and normal organoids (42 samples in total). Gene expression values were normalized and log2‐transformed [log2(TPM/FPKM +1)] [14]. Methylation data were initially curated by filtering out probes based on detection *p*‐values [15]. Analysis was confined to promoter‐associated genomic regions, including TSS1500, TSS200, 5′UTR, and the 1st Exon [16]. To evaluate the relationship between promoter methylation and gene expression, the mean *β* value across all probes located within a gene's promoter region was computed as a summary metric [17]. Comparative analysis of gene expression and methylation patterns between GIM and normal tissue samples was conducted utilizing the *limma* package in R [18]. Genes meeting the criteria of |log2(Fold Change)| > 1 with an adjusted *p*‐value < 0.05 were classified as differentially expressed (DEGs) [19]. Differentially methylated genes (DMGs) were identified using an adjusted *p*‐value < 0.05 and an absolute mean Beta difference (|Δ*β*|) > 0.1, corresponding to a 10% change in methylation level [20].

### 2.2 Identification of Epigenetically Dysregulated Genes and Transcription Factor Prediction

Genes whose promoter region methylation levels are negatively correlated with gene expression in GIM are identified as candidate epigenetic driver genes [21]. The 40 genes with low methylation levels in their promoter regions and high gene expression levels were subjected to transcription factor (TF) enrichment analysis applying the Enrichr platform (https://maayanlab.cloud/Enrichr/) [22] with the TRRUST database [23] to predict potential upstream regulators.

### 2.3 Single‐Cell RNA Sequencing Analysis of Gastric Organoids

scRNA‐seq data from 22 human gastric organoid samples (including undifferentiated and differentiated organoids from normal and GIM patients) were analysed. Initial processing of raw single‐cell RNA sequencing counts was performed with Seurat (version 5.0) [24]. Low‐quality cells expressing fewer than 200 genes and genes detected in less than 3 cells were removed. Normalization was achieved using the LogNormalize method, applying a scale factor of 10,000 [25]. The 2000 most variable genes were identified via the “vst” selection method [26]. Following data scaling and principal component analysis (PCA), the Harmony algorithm was implemented on the first 30 principal components to mitigate inter‐sample batch effects [27]. UMAP visualization and graph‐based clustering were performed on the Harmony‐corrected embeddings [28]. To investigate early lineage commitment, we subset the undifferentiated organoid cells (Normal vs. GIM) for downstream analysis. Cell identities were assessed utilizing module scores for: *Stemness* (*LGR5*, *OLFM4*, *ASCL2*, *PROM1*), *Gastric Identity* (*GKN1*, *TFF1*, *MUC5AC*), and *Intestinal Identity* (*CDX2*, *KRT20*, *MUC2*) [29]. Cell cycle phase was assigned using the *CellCycleScoring* function [30]. Final annotations integrated cluster‐specific marker genes (identified by *FindAllMarkers*), lineage module scores, and sample composition. Cell type proportions were compared between groups using stacked bar plots. Gene expression correlations were calculated employing Spearman's method [31].

### 2.4 Inclusion and Exclusion Criteria and Sample Sources of GIM Patients

Three subjects exhibiting features of GIM were enrolled in this study. The inclusion criteria did not restrict the characteristics of the lesions, which could be either focal or diffuse, nor the pathological subtype, which could be complete or incomplete. GIM was diagnosed based on the presence of glands containing goblet cells observed in haematoxylin and eosin (H&E) stained sections. *Helicobacter pylori* infection status was not applied for an inclusion or exclusion criterion, as infection may become undetectable following the development of metaplasia. Exclusion criteria encompassed individuals with hereditary predispositions like autosomal dominant diffuse gastric cancer. Table S1 contains comprehensive clinical data for the enrolled patients. All gastric tissue specimens were procured from the endoscopy suite at The Fifth Affiliated Hospital of Zhengzhou University. Ethical approval for this research was granted by the same institution's Ethics Committee (Approval No. KY2023123), with written informed consent acquired from every participant. GIM and adjacent normal gastric mucosa tissues were acquired from biopsy forceps. Each tissue was divided into two parts: one portion was fixed in 4% paraformaldehyde (PFA) for histopathological examination, the other was used for subsequent organoid culture experiments.

### 2.5 The Culture of Organoids

The endoscopic biopsies of gastric tissue were transferred into organoid basic medium. The tissues were washed twice with cold PBS containing penicillin/streptomycin (15140122, Gibco, USA) and gentamycin/amphotericin B (15750060, Gibco, USA). Collagenase type II was added for digestion after PBS was removed, and the mixture was shaken at 37°C for 30–40 min. Cold organoid basal medium supplemented with fetal bovine serum (FBS) (16140071, Gibco, USA) was introduced to terminate the digestion. The tube containing the suspension was centrifuged at 4°C and 1200 rpm for 5 min. The supernatant was discarded, and medium with FBS was added before passing the suspension through a 1 mL syringe 4–5 times to obtain single cells. The suspension was centrifuged again under the same settings; the supernatant was removed, and an appropriate amount of matrigel (082755, Mogengel‐Bio, China) was added. The cell‐matrigel mixture was seeded into a 24‐well plate and placed in the cell culture incubator for 10–15 min to allow the matrigel to solidify. 500 μL of gastric epithelial organoid medium (MA‐0817H003LP, Mogengel‐Bio, China) was added to each well for cultivation. The medium was replaced twice weekly and organoids were passaged roughly every 7–10 days [32].

### 2.6 The Culture of GES‐1 Cells

The human gastric epithelial cell line GES‐1 (SE‐C12345, sourced from Wuhan Shang'en Biotechnology, China) was cultured in RPMI‐1640 medium (Gibco, USA, 11875093) supplemented with 10% fetal bovine serum (FBS) and 1% penicillin/streptomycin. Cultures were kept at 37°C in a humidified atmosphere containing 5% CO_2_. Medium was changed every 48–72 h, and cells were dissociated with 0.25% trypsin–EDTA solution (Gibco, USA, 25200056) upon reaching 80%–90% confluence for subculturing [33]. Cell morphology and growth conditions were routinely monitored under an inverted microscope (model IX73, Olympus Corporation, Japan) to ensure healthy proliferation [34].

### 2.7 *H. pylori* Infection of GES‐1 Cells

The *H. pylori* strain SS1 was cultured on Columbia agar plates supplemented with 7% defibrinated sheep blood under microaerophilic conditions (85% N_2_, 10% CO_2_, and 5% O_2_) at 37°C for 48 h. Prior to infection, *H. pylori* was harvested and suspended in antibiotic‐free RPMI‐1640 medium, and the bacterial concentration was adjusted to an optical density of 0.1 at 490 nm (OD_490_). GES‐1 cells were seeded in 6‐well plates at a density of 3 × 10^5^ cells/well and cultured overnight until reaching 70%–80% confluence. The culture medium was then replaced with fresh antibiotic‐free RPMI‐1640 medium, and *H. pylori* was added at a multiplicity of infection (MOI) of 100:1 [35]. The cells were co‐cultured for 12 h at 37°C under microaerophilic conditions. Following co‐culture, the cells were washed three times with PBS to remove non‐adherent bacteria, and then harvested for subsequent experiments [36].

### 2.8 Immunohistochemical (IHC) Staining

Paraffin‐embedded human gastric mucosal tissue sections, including GIM and adjacent normal gastric tissues, were cut into 4 μm slices and mounted on glass slides. Following deparaffinization in xylene and rehydration through a descending ethanol gradient, tissue sections underwent heat‐induced epitope retrieval in citrate buffer via microwave irradiation. Endogenous peroxidases were inactivated by a 10‐min treatment with 3% aqueous hydrogen peroxide at room temperature [37]. Staining was executed with a commercial immunohistochemistry kit (PV‐9000, Zhongshan Jinqiao Biotechnology, China) per the supplied protocol. After blocking, sections were incubated at 4°C overnight with primary antibodies targeting pSTAT3 (Abcam, USA, ab76315) and CCND2 (Abcam, USA, ab226972) [38]. Subsequent steps included application of the kit's enhancer and secondary antibody, colour development with 3,3′‐diaminobenzidine (DAB), and nuclear counterstaining with haematoxylin. Two independent, blinded pathologists evaluated staining intensity and distribution [39].

### 2.9 Western Blot

GES‐1 cells were harvested before and after *H. pylori* infection, washed twice with ice‐cold PBS, and lysed on ice for 30 min in RIPA lysis buffer (Beyotime, China, P0013B) supplemented with 1% protease inhibitor cocktail (Roche, Switzerland, 04693159001) and 1% phosphatase inhibitor cocktail (Roche, Switzerland, 04906845001). Cell lysates were centrifuged at 12,000 × g for 15 min at 4°C, and the supernatants were collected. Protein concentrations were determined using a BCA protein assay kit (Thermo Fisher Scientific, USA, 23225). Equal amounts of protein (30 μg per lane) were separated by 10% SDS‐PAGE and subsequently transferred onto PVDF membranes (Merck Millipore, Germany, IPVH00010). Membranes were blocked with 5% non‐fat dry milk (BD Biosciences, USA, 232100) in TBST (Tris‐buffered saline containing 0.1% Tween‐20) for 1 h at room temperature, followed by overnight incubation at 4°C with primary antibodies diluted in blocking buffer. The primary antibodies used were as follows: anti‐CagA (Santa Cruz Biotechnology, USA, sc‐28368, 1:500), anti‐STAT3 (Cell Signalling Technology, USA, #9139, 1:1000), anti‐pSTAT3 (Abcam, USA, ab76315, 1:500), anti‐CCND2 (Abcam, USA, ab226972, 1:1000), and anti‐β‐actin (Proteintech, China, 66009‐1‐Ig, 1:5000) as a loading control. After three washes with TBST, the membranes were incubated with HRP‐conjugated goat anti‐rabbit or anti‐mouse secondary antibodies (1:5000; Cell Signalling Technology, USA, #7074 and #7076) for 1 h at room temperature. Protein bands were visualized using an enhanced chemiluminescence (ECL) detection kit (Thermo Fisher Scientific, USA, 34580) and imaged with a chemiluminescence imaging system (Tanon Science & Technology, China, Tanon‐5200). Band intensities were quantified using ImageJ software (National Institutes of Health, USA) and normalized to the corresponding β‐actin signals.

### 2.10 Dual‐Luciferase Reporter Assay

To assess STAT3‐dependent transcriptional activation of the *CCND2* gene, a luciferase reporter construct containing the human *CCND2* promoter region was generated in the pGL3‐basic vector (Promega, USA, E1751). GES‐1 cells were co‐transfected with this reporter plasmid along with either a STAT3 expression vector or its corresponding empty control, using Lipofectamine 2000 transfection reagent (Thermo Fisher Scientific, USA, 11668019) [40]. Transfection efficiency was monitored by co‐transfection of a Renilla luciferase control plasmid (pRL‐TK, Promega, USA, E2241) [41]. Luciferase activities were quantified 48 h post‐transfection using a dual‐luciferase assay system (Promega, USA, E1910). Firefly luminescence readings were normalized to Renilla values, and results from three independent experiments were expressed as relative luciferase activity [42].

### 2.11 Quantitative Real‐Time PCR (qRT‐PCR)

RNA isolation from human gastric organoids (derived from both normal and metaplastic mucosa) was performed with the RNeasy Mini Kit (Qiagen, Germany, 74106). RNA quality and yield were verified spectrophotometrically (NanoDrop, Thermo Fisher Scientific, USA) [43]. Reverse transcription of 1 μg total RNA was carried out using the ReverTra Ace qPCR RT Kit (TOYOBO, Japan, FSQ‐101). Quantitative PCR was conducted on a QuantStudio 5 instrument (Applied Biosystems, USA) with TB Green Premix Ex Taq II (Takara, Japan, RR820A) [44]. Transcript levels of *CDX2*, *MUC2*, *STAT3*, and *CCND2* were normalized to *GAPDH*, and relative quantification was determined via the 2^−ΔΔCT^ method, with each sample assayed in triplicate [45].

### 2.12 Chromatin Immunoprecipitation Followed by Quantitative PCR

Chromatin immunoprecipitation (ChIP) assays were performed as previously described [46]. Briefly, cells were cross‐linked with 1% formaldehyde for 10 min at room temperature to stabilize protein‐DNA interactions, and the reaction was quenched with 125 mM glycine. Cells were harvested, washed with ice‐cold PBS, and lysed in cell lysis buffer (5 mM PIPES pH 8.0, 85 mM KCl, 0.5% NP‐40) supplemented with a protease inhibitor cocktail (Roche, Switzerland, 4693132001). Nuclei were pelleted and resuspended in nuclear lysis buffer (50 mM Tris–HCl pH 8.0, 10 mM EDTA, 1% SDS). Chromatin was sheared to fragments of 200–600 bp using a Bioruptor Pico (Diagenode, Belgium, B01060010). After centrifugation, the soluble chromatin was diluted 10‐fold in ChIP dilution buffer and pre‐cleared with protein A/G magnetic beads (Thermo Fisher Scientific, USA, 26162). Immunoprecipitation was carried out overnight at 4°C using an anti‐pSTAT3 (Tyr705) antibody (Cell Signalling Technology, USA, #9145) or normal rabbit IgG (Cell Signalling Technology, USA, #2729) as a negative control. Protein A/G magnetic beads were added and incubated for an additional 2 h at 4°C. The beads were then washed sequentially with low‐salt, high‐salt, and LiCl wash buffers, followed by TE buffer. Protein‐DNA complexes were eluted, and cross‐linking was reversed by overnight incubation at 65°C in the presence of 200 mM NaCl. DNA was purified using a PCR purification kit (QIAGEN, Germany, 28106) and subjected to qRT‐PCR. qRT‐PCR was performed according to the above description. The enrichment of specific DNA fragments was calculated as a percentage of input DNA. Primers used for ChIP‐qPCR are listed in Table S2.

### 2.13 Immunofluorescence (IF) Staining

Organoids generated from normal and GIM gastric epithelia were fixed in 4% paraformaldehyde at 4°C overnight, then thoroughly washed with PBS. Specimens were permeabilized using 0.3% Triton X‐100 in PBS for 30 min and blocked with 5% bovine serum albumin (BSA) for 1 h at room temperature. Incubation with primary antibodies (anti‐CDX2 ab76541, anti‐MUC2 ab308191, anti‐pSTAT3 ab76315, anti‐CCND2 ab226972, anti‐SOX9 ab185966, and anti‐LGR5 ab273092; all from Abcam, USA) proceeded overnight at 4°C. After PBS washes, organoids were exposed for 1 h to Alexa Fluor 488‐conjugated anti‐rabbit (ab150077) and Alexa Fluor 594‐conjugated anti‐mouse (ab150116) secondary antibodies (Abcam, USA) under light‐protected conditions. Nuclei were visualized with DAPI (Beyotime, China, C1005) [47]. Finally, samples were mounted with anti‐fade medium (Beyotime, China, P0126) and visualized under a Leica TCS SP8 fluorescence microscope. Images were captured under identical settings to allow comparison among normal gastric epithelial, GIM organoids, and GIM treated with a STAT3 knocked‐down plasmid organoids group.

### 2.14 Statistical Analysis

Bioinformatic analyses were conducted in R software (v4.1.2) and *p* < 0.05 was considered statistically significant [48]. Experimental data, presented as mean ± SD, were analysed with GraphPad Prism 10 (GraphPad, USA) applying student's *t*‐test as two separate groups of numerical data approximately satisfied the assumptions of normal distribution and homogeneity of variance [49]. The asterisks were employed to indicate statistical significance, with n.s. means no significance, * denoting *p* < 0.05, ** indicating *p* < 0.01, *** representing *p* < 0.001 and **** corresponding to *p* < 0.0001.

## 3 Results

### 3.1 Genes Regulated by Promoter Methylation Were Identified in GIM

**FIGURE 1:**
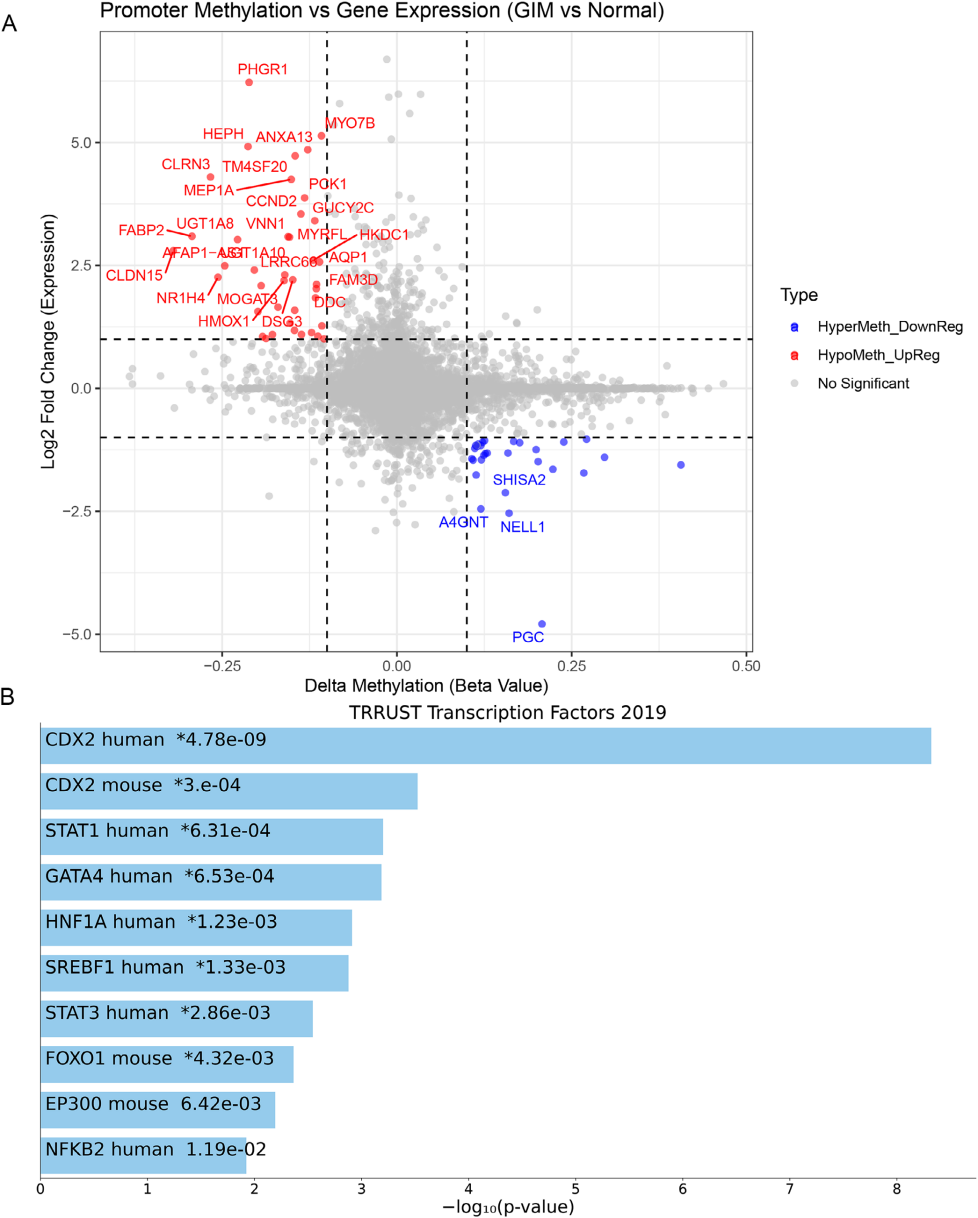
Promoter methylation‐transcriptomic integration and genes associated key TFs analysis in normal and GIM organoids. (A) The relationship between promoter methylation (Δ*β*, *x*‐axis) and gene expression (log2FC, *y*‐axis) in normal and GIM organoids were showed by Starburst plot. Dots represented individual genes. Red dots mean the 40 genes were significantly upregulated and the promoter regions were hypomethylated (Hypo‐Up) in GIM (adjusted *p* < 0.05, |Δ*β*| > 0.1, log2FC > 1). Blue dots signified the 29 genes were markedly downregulated and the methylation levels in their promoter regions were elevated (Hyper‐Down; adjusted *p* < 0.05, |Δ*β*| > 0.1, log2FC < −1). The remaining genes were shown as grey dots. (B) Top 10 TFs were enriched in the 40 upregulated genes in GIM, as predicted by TRRUST enrichment analysis through the Enrichr platform. The *y*‐axis showed the significance of enrichment. The smaller the value after the asterisk, the higher the enrichment level of the TF.

To identify the genes regulated by DNA methylation in GIM, we performed a joint analysis of promoter DNA methylation and gene expression in paired normal and GIM organoids (Table S3). A Starburst plot visualizing the relationship between methylation change (Δ*β*) and expression change (log2FC) revealed that the expression of 69 genes was negatively correlated with the methylation levels of their promoter regions, and the correlation was statistically significant in the paired organoids mentioned above (*p* < 0.05; Figure 1A). Among these, a cohort of 40 genes exhibited promoter hypomethylation coupled with transcriptional upregulation (Figure 1A, red dots), representing candidates for aberrant activation in GIM. Conversely, 29 genes were hypermethylated and downregulated (Figure 1A, blue dots), suggesting epigenetic silencing. In general, DNA methylation inhibits the binding of TFs, thereby downregulating gene expression [12]. To further analyse the transcriptional regulatory network controlling the upregulated gene signature in GIM, we performed TFs enrichment analysis targeting the upregulated genes in GIM. This analysis indicated the intestinal initial regulator CDX2 as the most significantly enriched TF among these genes, followed closely by a member of the STAT family, STAT1. Besides, STAT3 is also significantly enriched (Figure 1B).

### 3.2 TFs Enrichment Implicated STAT3 as a Key Upstream Regulator

**FIGURE 2:**
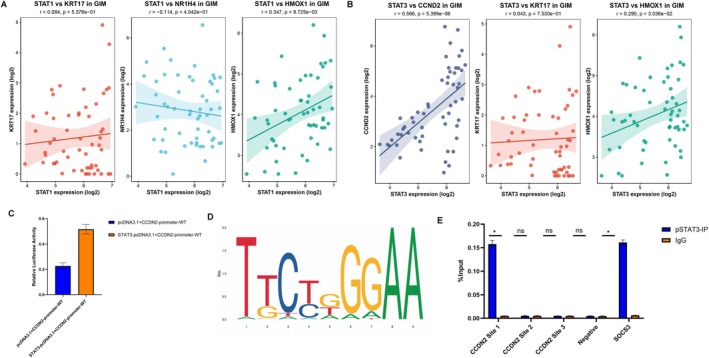
The correlation analysis of *STAT1* and *STAT3* with these related target genes and causal verification. (A, B) The correlation between the expression of transcription factors (*STAT3* or *STAT1*) and their predicted target genes within the GIM organoid transcriptome dataset was exhibited through scatter plots. Each dot represented a GIM organoid sample. The Spearman correlation coefficient (*r*) and corresponding *p*‐value were also displayed. (C) The Luciferase activity of the *CCND2* promoter reporter construct was shown upon co‐transfection with either an empty vector (pcDNA3.1) or a *STAT3* overexpression plasmid (STAT3‐pcDNA3.1) in GES‐1 cells. Firefly luciferase activity was normalized to Renilla luciferase. (D) Schematic diagram of the human CCND2 promoter region showing three putative pSTAT3 binding sites (site 1: 1078–1086 bp, TSS –923 bp; site 2: 49–57 bp, TSS –1952 bp; site 3: 526–534 bp, TSS –1475 bp) predicted by the JASPAR database. A negative control region (NC, 1050–1300 bp) without any predicted pSTAT3 motif is also indicated. The well‐established pSTAT3 binding site in the SOCS3 promoter serves as a positive control (PC). (E) ChIP‐qPCR analysis of pSTAT3 enrichment at the indicated regions of the CCND2 promoter in GIM organoids. Significant enrichment is detected only at site 1, comparable to the positive control SOCS3 promoter, while no enrichment is observed at sites 2, 3, or the negative control region. Data are shown as fold enrichment over IgG control (mean ± SEM); * represented *p* < 0.05, **p < 0.01, n.s. = not significant.

Given that CDX2 is already well recognized as the initiating TF of GIM, we selected STAT family members, STAT1 and STAT3, whose enrichment ranks immediately after CDX, for further investigation. The correlation between *STAT1* and *STAT3* and their related target genes in the GIM organoid transcriptome data was performed (Table S4). Notably, a strongest positive correlation was observed between *STAT3* and its predicted target *CCND2* (*r* = 0.566, *p* = 5.399e‐06; Figure 2B). In contrast, the correlations for *STAT1* with its predicted targets were substantially weaker compared to *STAT3* (Figure 2A). Accordingly, the STAT3‐CCND2 axis served as a candidate driver mechanism for further functional validation. To determine whether STAT3 can act on the *CCND2* promoter region to promote its transcription, the dual‐luciferase reporter assay was conducted. It was revealed that co‐transfection of a *STAT3* overexpression plasmid with a luciferase construct driven by the human *CCND2* promoter resulted in a significant increase in promoter activity compared to the empty vector control (Figure 2C). This result provided direct in vitro evidence that STAT3 could bind to the promoter region of *CCND2* to enhance its transcription.

To further confirm whether pSTAT3 directly binds to the *CCND2* promoter in the context of chromatin, ChIP‐qPCR was performed. Based on JASPAR database predictions, three putative pSTAT3 binding sites within the CCND2 promoter region were selected: site 1 (1078–1086 bp, TSS −923 bp), site 2 (49–57 bp, TSS −1952 bp), and site 3 (526–534 bp, TSS −1475 bp; Figure 2D). A negative control region (1050–1300 bp) lacking any predicted pSTAT3 binding motif was also included, and the well‐established pSTAT3 binding site within the *SOCS3* promoter served as a positive control. The ChIP‐qPCR results demonstrated that pSTAT3 was significantly enriched only at site 1 of the *CCND2* promoter compared to the IgG control, with an enrichment level comparable to that observed at the positive control *SOCS3* promoter (Figure 2E). In contrast, no significant enrichment was detected at sites 2, 3, or the negative control region, indicating that pSTAT3 binds specifically to a defined site within the CCND2 promoter to drive its transcription (Figure 2E). These findings collectively confirm that pSTAT3 directly binds to the *CCND2* promoter and transcriptionally activates *CCND2* expression, establishing the STAT3‐CCND2 axis as a direct transcriptional regulatory module in GIM pathogenesis.

### 3.3 Cellular Heterogeneity Was Revealed Through Single‐Cell Transcriptomic Atlas of Undifferentiated Gastric Organoids

**FIGURE 3:**
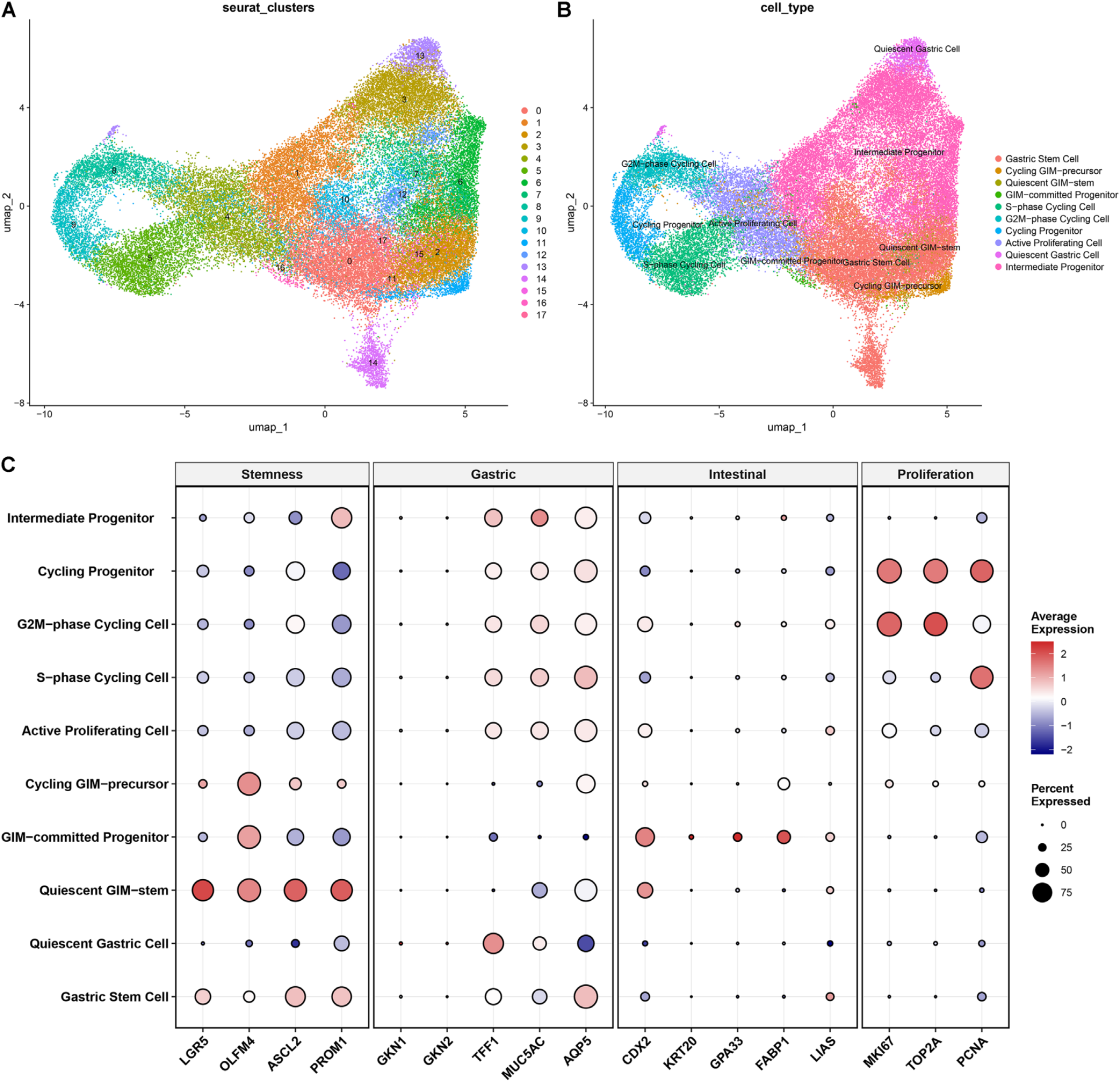
The single‐cell transcriptomic landscape of undifferentiated gastric organoids. (A) The UMAP visualization of 49,941 undifferentiated cells from normal and GIM gastric organoids after Harmony integration was coloured by unsupervised graph‐based clustering (18 clusters). (B) The UMAP plot was coloured based on annotated cell type identity determined by integrating cluster‐specific markers, lineage module scores and cell cycle phase. (C) The expression and cell percentages expressing key marker genes across the 10 annotated cell types were displayed through dot plot. The redder the colour, the higher the gene expression level; the bluer the colour, the lower the gene expression level. The larger the dot, the higher the proportion of cells expressing that gene.

To dissect the cellular landscape of early GIM commitment at high resolution, a single‐cell transcriptomic atlas from undifferentiated gastric organoids was generated. 18 transcriptionally distinct cell clusters were revealed by unsupervised clustering following batch correction (Figure 3A). 10 major cellular states were distinguished through annotation based on gene module scores for stemness, gastric identity, and intestinal identity, alongside cell cycle analysis (Figure 3B; Table S5 and Figure S1). Specifically, gastric stem cells, various proliferating progenitor populations (cycling progenitor and S/G2M‐phase cells) and GIM‐committed lineages, such as cycling GIM‐precursor, were included (Figure 3C). The atlas provided a foundational resource for investigating lineage‐specific regulatory programs.

### 3.4 GIM Organoids Were Dominated by a Hyperproliferative, GIM‐Committed Progenitor Cells

**FIGURE 4:**
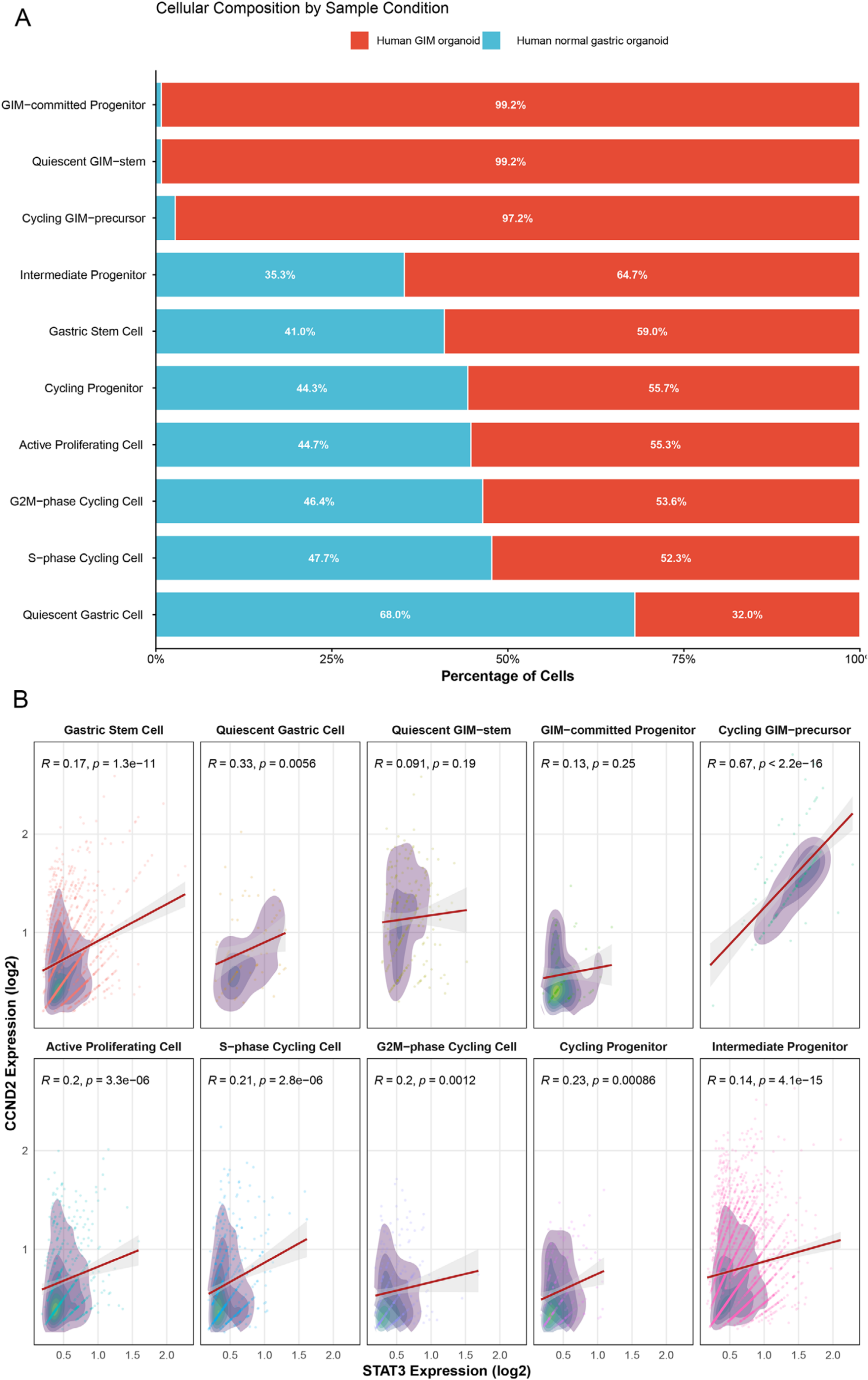
The cellular composition of human GIM and normal gastric organoids and the correlation between the expression of *STAT3* and *CCND2* in different cells. (A) The proportional composition of major cell types between undifferentiated normal (*n* = 19,492 cells) and GIM (*n* = 30,449 cells) organoids was compared by stacked bar chart. (B) The expression distribution of *STAT3* and *CCND2* across key cell types was shown through Violin plots. The Spearman correlation coefficient (*R*) and corresponding *p*‐value were also displayed.

Comparative analysis unveiled a dramatic shift of cellular composition between normal and GIM organoids. Normal organoids were predominantly composed of gastric stem cells and quiescent lineages. While GIM organoids showed a marked expansion of proliferative and GIM‐fated populations (Figure 4A). Specifically, clusters such as cycling GIM‐precursor (Cluster 11) and GIM‐committed progenitor (Cluster 16) were mainly derived from GIM samples (> 95% GIM cells), indicating the emergence and maintenance of a distinct, primed progenitor compartment specific to the metaplastic state. Given the hyperproliferative phenotype of GIM organoids and our prior identification of the STAT3‐CCND2 axis, we analysed their co‐expression patterns at the single‐cell level. Spearman correlation analysis demonstrated a significant positive correlation between *STAT3* and *CCND2* expression across multiple cell types (Figure 4B).

### 3.5 pSTAT3 and CCND2 Are Progressively Upregulated Across the Correa Cascade and Induced by *H. pylori* Infection

**FIGURE 5:**
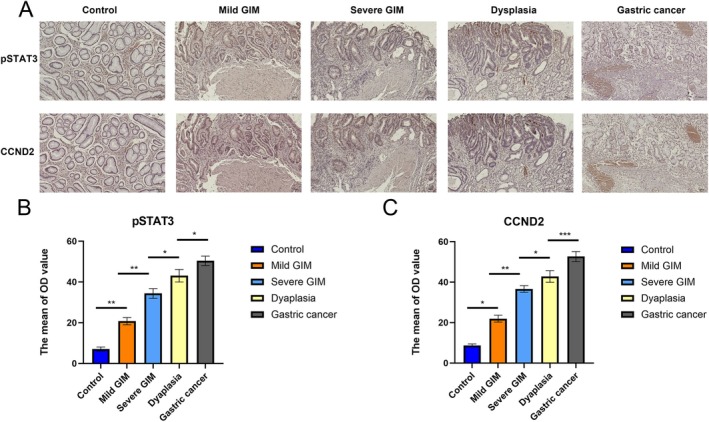
The changes of pSTAT3 and CCND2 in the normal gastric and GIM mucosa tissues. (A) The expression of pSTAT3 and CCND2 in the control, mild GIM, severe GIM, dysplasia, and GC group. A deeper brown coloration in the images indicates higher expression levels of the target protein, while a larger brown‐stained area reflects a broader distribution of the protein expression. (B, C) The bar graph visualization analysis of pSTAT3 and CCND2 expression in the above tissue sections. *p < 0.05; **p < 0.01; ***p < 0.001.

**FIGURE 6:**
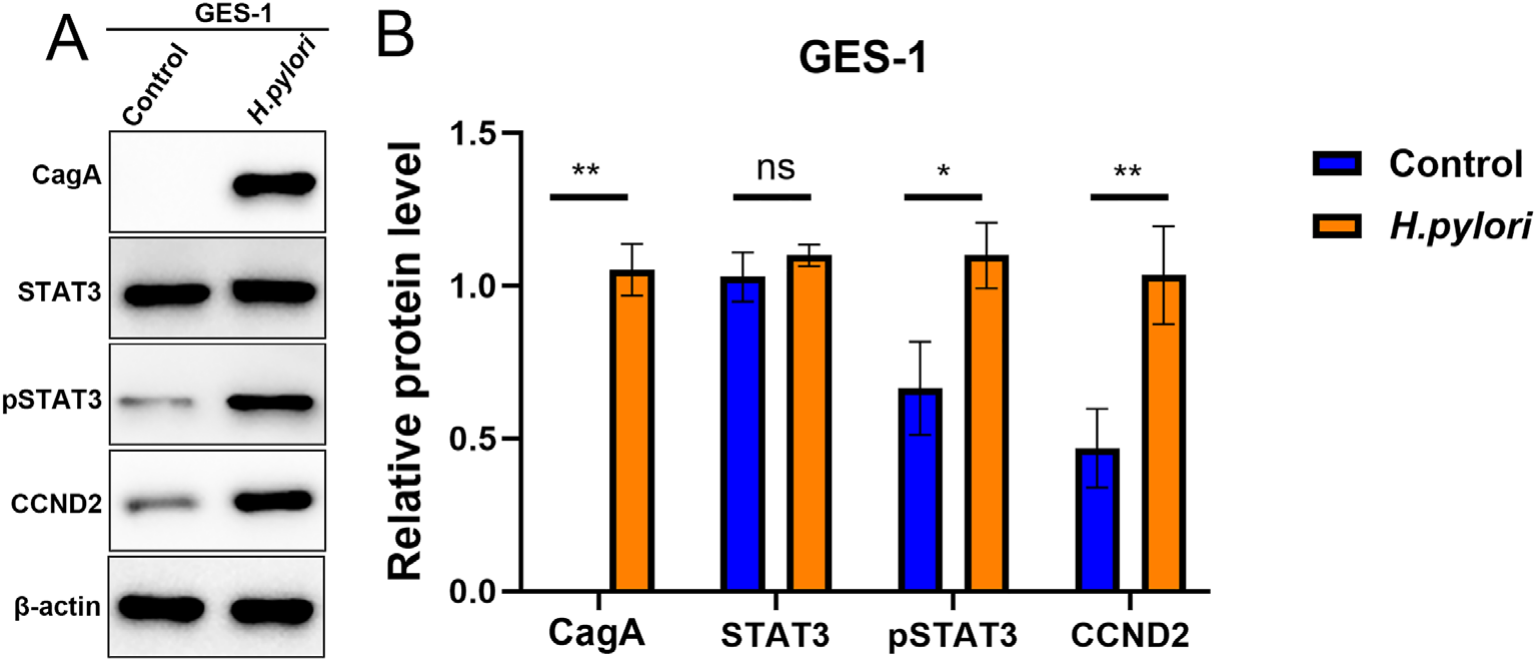
The expression of pSTAT3 and CCND2 in GES‐1 cells before and after *H. pylori* infection. (A) The expression of CagA, STAT3, pSTAT3, and CCND2 in GES‐1 cells before and after *H. pylori* infection. (B) The visualization analysis of the expression of the above proteins in GES‐1 cells before and after *H. pylori* infection. n.s., no significance; *p < 0.05; **p < 0.01.

To further validate the expression patterns of STAT3 and CCND2 in gastric carcinogenesis, IHC was performed on human tissue sections spanning the entire Correa cascade, including normal gastric mucosa, mild GIM, severe GIM, dysplasia, and GC. The results demonstrated that both pSTAT3 and CCND2 exhibited progressively increased expression levels along the histological spectrum (Figure 5A). The differences between adjacent stages were all statistically significant (Figure 5B,C). Moreover, since *H. pylori* is the most important and common pathogenic factor causing GIM [50], and CagA is the most prevalent virulence factor of its standard strain SS1, we further explored the regulatory relationship between *H. pylori* infection and these two proteins in GES‐1 cells by western blot. A marked increase in both pSTAT3 and CCND2 was detected in *H. pylori‐*infected GES‐1 cells (Figure 6A,B), indicating that *H. pylori* triggers the activation of the STAT3 pathway and upregulation of CCND2. Collectively, these findings indicate that the STAT3‐CCND2 axis is progressively activated during the Correa cascade and can be induced by *H. pylori* , supporting their potential roles as biomarkers and therapeutic targets in the progression from GIM to GC.

### 3.6 *STAT3* Positively Regulated the Expression of *CCND2* , *CDX2* and *MUC2* in Gene Level

**FIGURE 7:**
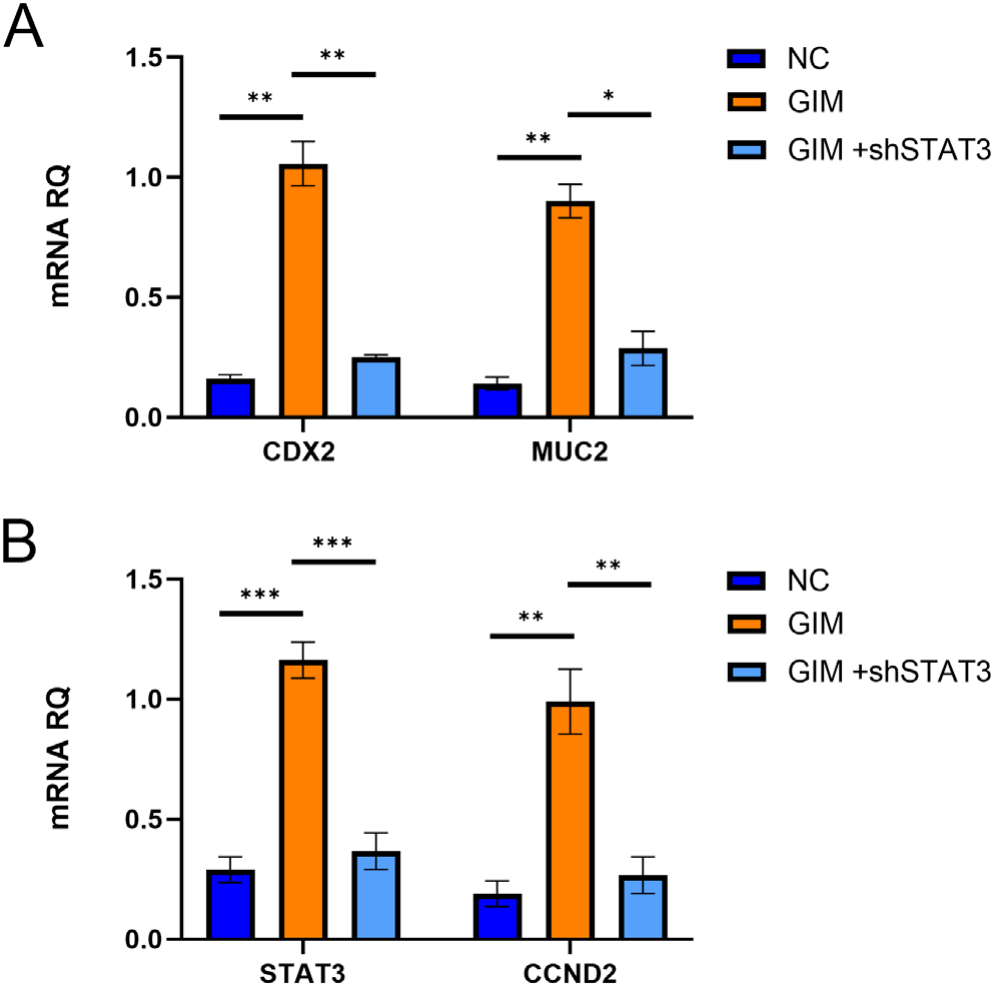
The *CDX2*, *MUC2*, *STAT3* and *CCND2* expression among normal, GIM and GIM knocked down *STAT3* groups (A, B). *p < 0.05; **p < 0.01; ***p < 0.001.

qRT‐PCR was performed on three pairs of normal and GIM gastric epithelial organoids to validate the expression levels of *STAT3*, *CCND2*, *CDX2*, and *MUC2*. The results showed that these genes were significantly upregulated in GIM organoids compared to controls. Furthermore, the knockdown of *STAT3* in GIM organoids led to a marked decrease in the expression of *STAT3*, *CCND2*, *CDX2*, and *MUC2* relative to untreated GIM organoids (Figure 7A,B). All observed differences were statistically significant, indicating that *STAT3* positively regulated the expression of these genes in GIM organoids.

### 3.7 STAT3 Promoted the Expression of CCND2 and GIM Markers and the Upregulation of Stemness Markers at the Protein Level

**FIGURE 8:**
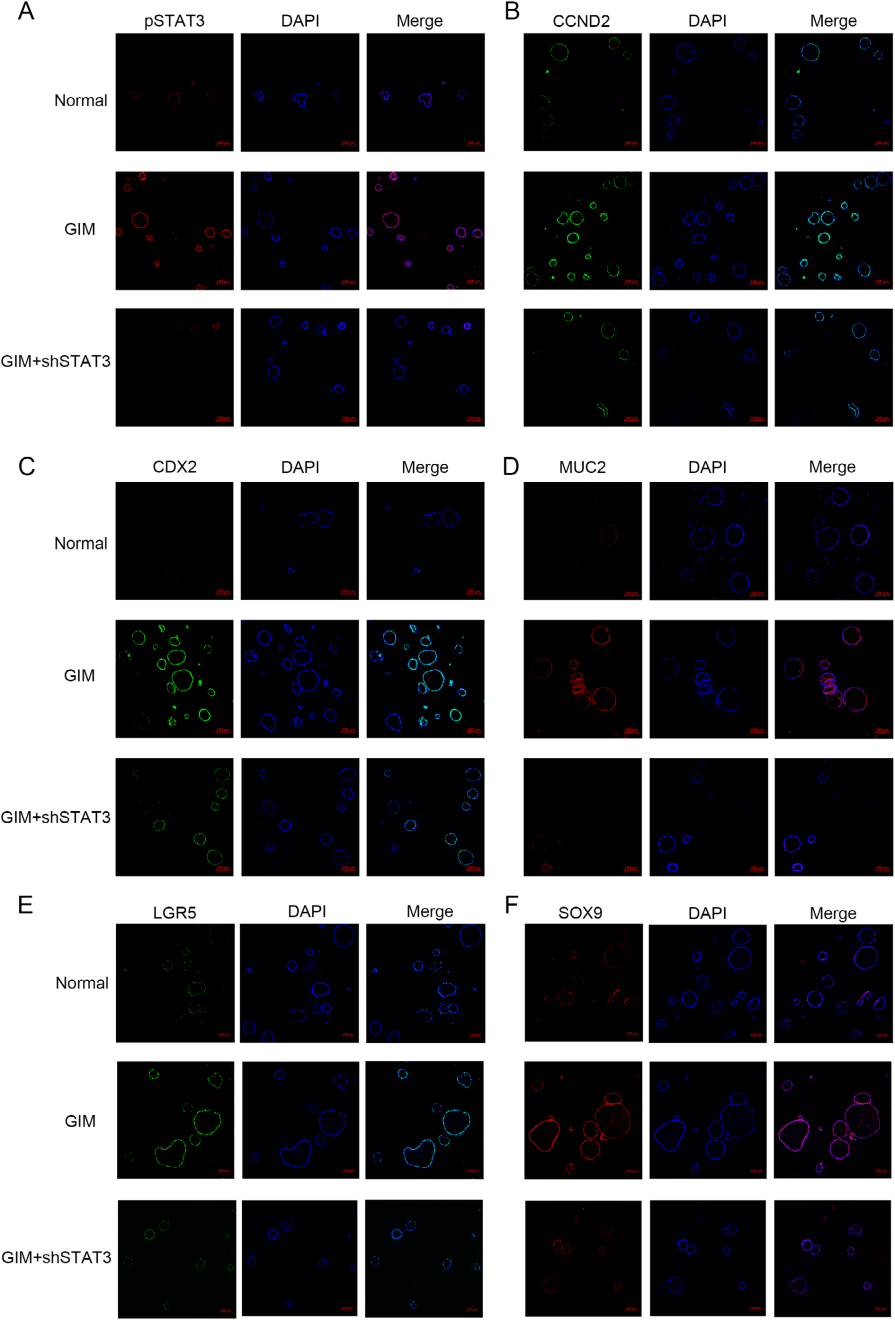
The expression of pSTAT3, CCND2, and GIM markers CDX2 and MUC2, as well as stemness markers LGR5 and SOX9 among normal, GIM, and GIM knocked down *STAT3* groups (A–F).

IF staining was performed on three pairs of control and GIM organoids to assess the protein expression of pSTAT3, CCND2, and the GIM markers CDX2 and MUC2, as well as the stemness markers LGR5 and SOX9. Consistent with previous findings, enhanced expression of pSTAT3, CCND2, CDX2, and MUC2 was observed in GIM organoids compared to controls. In parallel, LGR5 and SOX9 were also significantly upregulated in GIM organoids relative to controls. Notably, *STAT3* knockdown in GIM organoids resulted in a substantial reduction in the expression of pSTAT3, CCND2, CDX2, MUC2, LGR5, and SOX9 compared with untreated GIM organoids (Figure 8A–F). These results further verified the critical role of *STAT3* in modulating the expression of these proteins during GIM progression and maintaining stemness properties.

## 4 Discussion

This study revealed the STAT3‐CCND2 axis in driving the initiation and progression of GIM through an integrated approach combining multi‐omics analysis, single‐cell transcriptomic mapping and systematic functional validation. Our work not only confirmed the co‐activation of *STAT3* and its target gene *CCND2* in GIM tissues, but also provided the first spatiotemporal resolution of its function: *STAT3*, by directly transactivating *CCND2*, specifically promoted the expansion of a proliferative ‘intestinal metaplasia‐precursor’ cell state during the early phase of gastric epithelial reprogramming, thereby supplying the crucial cellular engine for the occurrence and maintenance of GIM. This finding offers a novel framework for understanding the cellular origins and molecular mechanisms of GIM, a critical precancerous lesion for gastric adenocarcinoma.

Traditionally, the role of *STAT3* in GC and its precursors has been largely confined to its functions in mediating inflammatory signalling, promoting cell survival and facilitating tumour immune evasion [51, 52, 53]. However, our study repositions and specifies its role earlier in the carcinogenic cascade. The integrative methylation‐transcriptomic analysis pointed *STAT3* as a master regulator of a gene set in which the promoter region was aberrantly hypomethylated and the corresponding gene was upregulated in GIM. Subsequent functional assays provided clear evidence that *STAT3* directly binds to the promoter of *CCND2* and promotes the transcription of *CCND2*. *CCND2*, a member of the cyclin D family, is a core switch driving cell cycle progression from G1 to S phase [54]. Previous studies have demonstrated a marked upregulation of *CCND2* expression in GIM relative to normal gastric mucosa [55]. Previous research in colorectal cancer has shown that *STAT3* directly binds to the *CCND2* promoter to drive its transcription, thereby supporting the maintenance and growth of persistent cancer stem cells (CSCs) and contributing to tumour progression [56]. Additionally, the STAT3‐CCND2 axis has also demonstrated a strong positive correlation with the development and progression of tumours such as thyroid adenoma [57], nasopharyngeal carcinoma [58], and lung adenocarcinoma [59]. Therefore, the establishment of the STAT3‐CCND2 axis directly couples a classic inflammatory/stress‐response pathway to the core cell cycle machinery. This offers a direct molecular explanation for the universal epithelial hyperproliferation observed in GIM lesions.

To further validate the clinical relevance of this axis along the Correa cascade, we examined pSTAT3 and CCND2 expression in human gastric tissues spanning normal mucosa, mild GIM, severe GIM, dysplasia, and gastric cancer. A progressive and statistically significant increase in both proteins was observed across this histological spectrum, with the highest expression detected in GC. These results indicate that the STAT3‐CCND2 axis is not only activated at the GIM stage but is continuously upregulated during gastric carcinogenesis, supporting its potential as a stage‐independent driver and a broad‐spectrum biomarker. Moreover, given that chronic *H. pylori* infection is a major initiating factor of the Correa cascade [60], we investigated whether *H. pylori* directly induces pSTAT3 and CCND2 in gastric epithelial GES‐1 cells. The expression levels of both pSTAT3 and CCND2 were markedly elevated after *H. pylori* infection in GES‐1 cells, demonstrating that this axis could be triggered by the primary environmental driver of GIM. Collectively, these tissue‐spectrum and infection experiments anchor the STAT3‐CCND2 axis firmly within the established aetiology and progression of GIM. Mechanistically, *H. pylori* is known to activate the JAK2/STAT3 signalling pathway as well as the β‐catenin/COX‐2 axis to induce intestinal differentiation during GIM pathogenesis [61, 62]. Our finding that *H. pylori* upregulates both pSTAT3 and CCND2 in gastric epithelial cells raises the possibility that *H. pylori* exerts its pro‐metaplastic effects at least in part through the STAT3‐CCND2 transcriptional module. Virulence factors such as CagA may enhance pSTAT3 recruitment to the CCND2 promoter, thereby driving CCND2 expression and subsequent cell cycle progression. Furthermore, crosstalk between the STAT3 pathway and other *H. pylori* ‐activated cascades (e.g., β‐catenin/COX‐2) could synergistically potentiate the STAT3‐CCND2 axis. Future studies using *H. pylori* ‐infected GIM organoids or STAT3/CCND2 perturbation models are warranted to dissect the causal role of this axis in *H. pylori* ‐induced gastric metaplasia.

Significantly, our findings demonstrate that *STAT3* activation concurrently upregulates key intestinal markers *CDX2* and *MUC2* [63]. This suggests that *STAT3* may act as a “conductor”, simultaneously accelerating cell proliferation through *CCND2* and co‐activating intestinal differentiation programs, thereby efficiently coordinating the large‐scale “reprogramming” of the gastric epithelial reservoir toward an intestinal phenotype cell [64, 65]. The coordinated activation of proliferation and differentiation programs likely constitutes a key mechanism enabling the rapid formation and stable maintenance of GIM.

One of the most insightful findings of this study originates from the single‐cell transcriptomic analysis of undifferentiated organoids. This atlas allowed us to move beyond “averaged” tissue signals and precisely identify the specific cellular populations altered during GIM development [66]. We observed a fundamental remodelling of cellular composition in GIM organoids compared to their normal counterparts: populations with gastric stem cell properties were relatively diminished, while a unique population of “cycling GIM‐precursors” characterized by high intestinal marker expression and an active cell cycle state was markedly expanded. The near absence of this population in normal samples strongly suggests it is a specific product of the GIM pathology.

Crucially, correlation analysis revealed that the co‐expression of *STAT3* and *CCND2* was most prominent within this cycling GIM‐precursor population. This spatially fixs the activity of this regulatory axis to the most proliferation‐competent transitional cell state. To functionally interrogate the role of STAT3 in maintaining stemness properties within the metaplastic context, we generated human GIM organoids with stable knockdown of *STAT3* and compared them to normal gastric organoids and control GIM organoids. The knockdown of *STAT3* led to a significant reduction in the stemness markers SOX9 [67] and LGR5 [68], indicating that *STAT3* is required for sustaining the progenitor‐like phenotype in GIM. This result directly links the STAT3‐CCND2‐proliferation axis to the maintenance of a stemness‐permissive state, further supporting the model that the cycling GIM‐precursor population is *STAT3*‐dependent. We may propose a dynamic model: under the persistent stimulus of initial pathogenic factors, such as chronic *H. pylori* infection, *STAT3* signalling becomes aberrantly activated within gastric epithelial stem or progenitor cells. These cells acquire a strong proliferative advantage via *CCND2* while initiating intestinal transcriptional programs like *CDX2*, thereby transforming into the defined cycling GIM‐precursor state driven by *STAT3*. This population acts as the ‘engine’ of metaplasia; its continuous proliferation and self‐renewal persistently produce more differentiated intestinalized epithelial cells, ultimately forming macroscopic GIM lesions. This model integrates molecular mechanisms (the STAT3‐CCND2 axis) with cell biological events (expansion of a specific precursor cell), providing a potential precision target for intervening in the early stages of GIM development.

Although this study focuses on the STAT3‐CCND2 axis itself, our data also point to more upstream regulation and broader downstream networks. The epigenomic analysis implies that components of the *STAT3* signalling pathway or its regulators might themselves be modulated by epigenetic modifications, such as hypomethylation, in the GIM microenvironment, predisposing them to a “pre‐activated” or easily activated state. Cytokines from the chronic inflammatory environment, such as IL‐6 family, classic activators of STAT3, and how they interact with epigenetic alterations to collectively activate this axis, represent an attractive direction for future inquiry.

## 5 Conclusion

In summary, this study systematically elucidates the central role of the STAT3‐CCND2 axis in GIM, showing that it is progressively activated along the Correa cascade, induced by *H. pylori* , and required for maintaining stemness in metaplastic lesions. This axis addresses the dual requirements of metaplasia: cell number and cell identity, through driving a proliferative precursor population as well as coordinating proliferation and differentiation. Our findings provide a foundation for future diagnostic and targeted interception strategies in gastric carcinogenesis.

## Author Contributions

**Simeng Liu:** funding acquisition, project administration, supervision. **Fazhan Li:** software, visualization, writing – review and editing. **Feifei Ren:** software, visualization. **Pengyuan Zheng:** data curation, supervision. **Huijuan Wen:** data curation, methodology, software, visualization, writing – original draft, writing – review and editing.

## Funding

This work was supported by the 2025 Henan Province Science and Technology Research Plan (No. LHGJ20250391), the 2024 Henan Province Overseas Training Program for Medical Science and Technology Talents, the 2025 International Science and Technology Cooperation Cultivation Program of Henan Province (No. 252102520089), and the 2025 Youth Student Basic Research Project of Zhengzhou University (No. ZDBJ202542).

## Ethics Statement

GIM and surrounding normal tissue specimens were obtained from the Fifth Affiliated Hospital of Zhengzhou University. The ethics committee of the Fifth Affiliated Hospital of Zhengzhou University approved the study (Ethics number: KY2023123).

## Consent

All the related patients have signed informed consent.

## Conflicts of Interest

The authors declare no conflicts of interest.

## Supporting information


**Figure S1:** Grouping and cell cycle analysis of normal and GIM organoids (A and B).

**Table S1:** The clinical information of 3 GIM patients.
**Table S2:** The Primers sequence of ChIP‐qPCR.
**Table S3:** The 69 screened genes.
**Table S4:** Correlations of transcription factors and their target genes.
**Table S5:** Cell annotation of single cells.

## Data Availability

The datasets analysed in this study were derived from the following sources. The primary multi‐omics and single‐cell RNA sequencing data for human gastric organoids were obtained from the Gene Expression Omnibus (GEO) under accession number GSE210995. Experimental validation was performed using the human gastric epithelial cell line GES‐1 and gastric tissues from GIM patients. Detailed experimental protocols, the processed data supporting the findings, and any other relevant materials are available from the corresponding author upon reasonable request.
