# Supplementary material for: The STAT3‐CCND2 Axis Drives a Proliferative Metaplastic Precursor Population in Gastric Intestinal Metaplasia
Source: J Cell Mol Med. 2026 Jul 15;30(14):e71283. doi: 10.1111/jcmm.71283 (PMC13370793; doi:10.1111/jcmm.71283)
Supplement: Supplementary file 1 — Figure S1: Grouping and cell cycle analysis of normal and GIM organoids (A and B). [file JCMM-30-e71283-s002.docx]

**Supplementary Figure 1:**

**
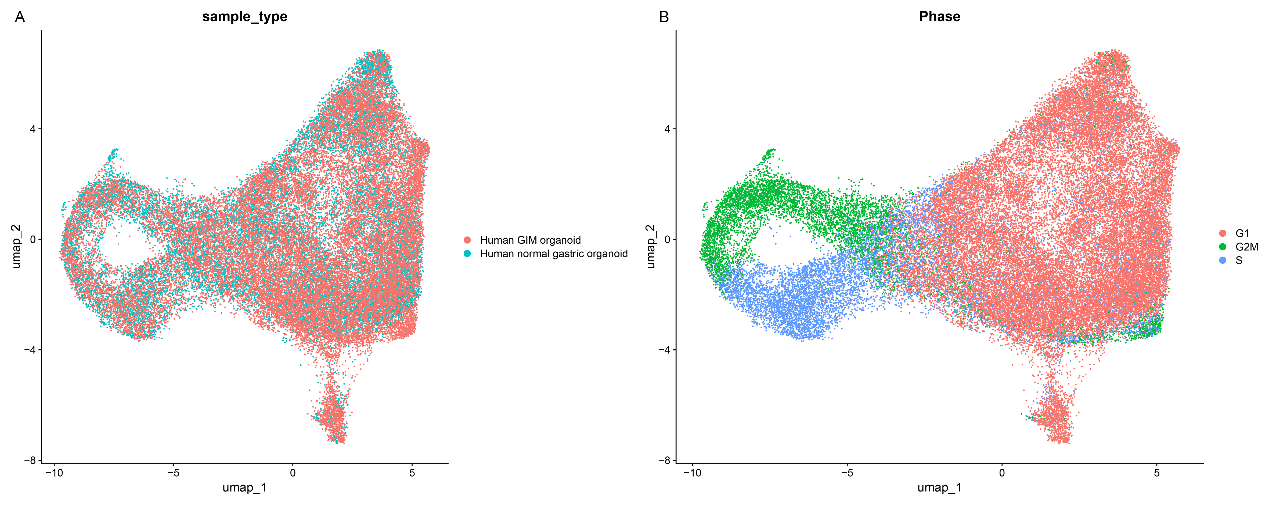
**

**Supplementary Figure 1: Grouping and cell cycle analysis of normal and GIM organoids (A & B).**
